# Refractory Immune Thrombocytopenic Purpura and Cytomegalovirus Infection: A Call for a Change in the Current Guidelines

**DOI:** 10.4084/MJHID.2016.010

**Published:** 2016-01-01

**Authors:** Alexei Shimanovsky, Devbala Patel, Jeffrey Wasser

**Affiliations:** 1 Department of Hematology and Oncology, University of Connecticut Health Center, 263 Farmington Ave, Farmington, CT 06030; 2 Department of Pathology and Laboratory Services, Manchester Memorial Hospital, 71 Haynes Street, Manchester, CT 06040

## Abstract

Immune thrombocytopenic purpura (ITP) is characterized by a decreased platelet count caused by excess destruction of platelets and inadequate platelet production. In many cases, the etiology is not known, but the viral illness is thought to play a role in the development of some cases of ITP. The current (2011) American Society of Hematology ITP guidelines recommend initial diagnostic studies to include testing for HIV and Hepatitis C. The guidelines suggest that initial treatment consist of observation, therapy with corticosteroids, IVIG or anti D. Most cases respond to the standard therapy such that the steroids may be tapered and the platelet counts remain at a hemostatically safe level. Some patients with ITP are dependent on long-term steroid maintenance, and the thrombocytopenia persists with the tapering of the steroids. Recent case reports demonstrate that ITP related to cytomegalovirus (CMV) can persist in spite of standard therapy and that antiviral therapy may be indicated. Herein we report a case of a 26-year-old female with persistent ITP that resolved after the delivery of a CMV-infected infant and placenta. Furthermore, we review the current literature on CMV-associated ITP and propose that the current ITP guidelines be amended to include assessment for CMV, even in the absence of signs and symptoms, as part of the work-up for severe and refractory ITP, especially prior to undergoing an invasive procedure such as splenectomy.

## Introduction

Immune thrombocytopenic purpura (ITP) is a common cause of acquired thrombocytopenia caused by auto-anti-platelet antibodies that destroy platelets, damage megakaryocytes and inhibit platelets production. Viral infection such as rubella, varicella, mumps, cytomegalovirus (CMV) and Epstein-Barr virus are linked to ITP and thrombocytopenia.^1^ While the infections can be relatively asymptomatic, they can trigger an autoimmune process. In the past several years, cases of ITP have been reported secondary to unsuspected, persistent infections such as hepatitis C, HIV and *Helicobacter pylori* (*H. pylori*)*.* The successful treatment of ITP in such cases may require recognition and eradication of the underlying infection.

Several cases of thrombocytopenia and ITP, secondary to persistent CMV infection, in immunocompetent adults, have been reported in the literature.^2^ While the majority of cases describe patients that responded to standard ITP therapy, some reports describe patients who are dependent upon or worsen with corticosteroids.^2^–^5^ In a small case-series of four patients, steroids appeared to worsen CMV-associated ITP and improvement in the platelet counts occurred after starting gancyclovir and Cytogam with steroid taper.^6^ Herein we describe a case of an immunocompetent adult female diagnosed with ITP, refractory to standard therapy, during her first pregnancy. The ITP improved following delivery of a CMV-infected neonate and the products of conception. The placenta demonstrated evidence of active CMV infection by PCR. Furthermore, we review current literature and propose a modification to the current ITP guidelines.

## Case Report

**Figure 1:**
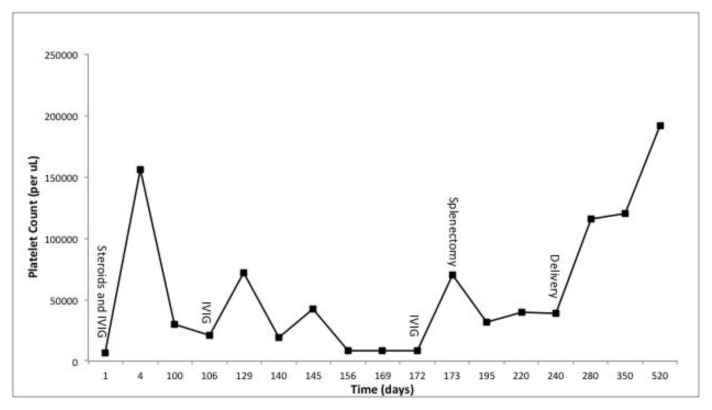
Patients’ clinical course. Time-points of administration of high-dose steroids, intravenous Immunoglobulin (IVIG), splenectomy and delivery of the infant are indicated. The patient was maintained on prednisone 40 mg PO daily until the day of delivery. After delivery, the prednisone was tapered off.

A healthy 26-year-old G1P0 female of Asian-Indian descent was referred to our clinic for a second opinion of her thrombocytopenia. During her initial visit to the obstetrician, she was asymptomatic and had a platelet count of 3 x 10^9^/l (normal 150-450 x 10^9^/l). She initially received intravenous immunoglobulin (IVIG) at 1 g/kg, prednisone 140 mg/day and a trial of anti-D. Her platelet count improved temporarily, returning to 10–30×10^9^//l after a few weeks (Figure 1). She was on maintenance prednisone (40 mg/day) when she first presented to our clinic.

The patient denied having an abnormal menstrual cycle, mucosal bleeding, and family history of blood disorders. On physical exam, the patient appeared cushingoid with normal vital signs. She had no petechiae or ecchymosis. Uterus was gravid. She had no palpable hepatosplenomegaly. The rest of her exam was normal. The leucocyte count was 24,500/uL, with 7% band forms, 88% neutrophils, and 4% lymphocytes. The hemoglobin was 13 g/dL with a hematocrit of 37%. Her platelet count was 30×10^9^/l. Coagulation screen, liver profile, and blood chemistry values were normal. Bone marrow biopsy showed megakaryocyte hyperplasia with normal morphology, consistent with ITP. Her ANA, Hepatitis B, C, and HIV were negative. Stool *H. pylori* antigen was positive.

The patient was treated with pantoprazole 40 mg daily, amoxicillin 1000 mg daily and clarithromycin 500 mg twice daily. After treatment, her platelet count remained between 10–40×10^9^//l. As resistance to *H. pylori* treatment is common, the patient received a second course of antibiotic therapy. Subsequently, the *H. pylori* stool antigen became negative with no improvement in platelet count. After receiving appropriate vaccinations, she underwent a splenectomy with no improvement in platelet count. She was maintained on prednisone 40 mg/day and intermittent IVIG until her delivery.

She delivered a healthy baby girl with a platelet count of 15×10^9^//l and no significant hemorrhage or bleeding. During a routine screen, the infant was diagnosed with congenital CMV via urine PCR. The newborn was treated with ganciclovir 10mg/kg, leading to improvement in platelet count. Placental pathology demonstrated focal chorionic chorionitis without any chorionic villinits. The corresponding segment of the placenta was positive for CMV by PCR. The mother was positive for CMV IgG. Interestingly, one week after delivery, the patient’s platelet count began to improve. She was no longer taking prednisone or IVIG and her platelet count normalized to 192×10^9^/l ten months after delivery (Figure 1).

## Discussion

Our patient presented with severe thrombocytopenia in her first trimester of pregnancy. While gestational thrombocytopenia accounts for 70–80% of cases during pregnancy, our patient did not meet the clinical criteria for this diagnosis. Her platelet count was less than 100 x 10^9^/l and she presented early in pregnancy, findings that are less consistent with gestational thrombocytopenia.^7^ While type 2B von Willebrand was considered in the differential of thrombocytopenia in our patient, it is less likely in our patient as she did not have any personal or family history of abnormal bleeding. Given the normal vital signs and liver function, pregnancy-specific causes of thrombocytopenia such as preeclampsia, eclampsia, HELLP syndrome and acute fatty liver are less likely. Moreover, the patient’s bone marrow was consistent with ITP and the temporary response to corticosteroids and IVIG supports the diagnosis of ITP.

ITP is a rare cause of thrombocytopenia during pregnancy, occurring only 1 in 10,000 pregnancies. While ITP can occur at any point in pregnancy, it is one of the few causes of thrombocytopenia that can become apparent during the first trimester.^8^ Primary ITP is caused by a complex mechanism that is caused by autoantibodies against platelets and T-cell mediated platelet destruction.^9^ Conversely, secondary ITP develops in a setting of autoimmune disease, lymphoma, and infection with *H. pylori* or viral infection such as HIV, hepatitis C, or CMV.^7^

The current ITP guidelines from the American Society of Hematology recommend testing for Hepatitis C and HIV, as they are known to cause secondary ITP.^10^ Both were negative in our patient. Testing for *H. pylori* is also recommended in patients with isolated thrombocytopenia. The stool antigen for *H. pylori* positive in our patient and we considered *H. pylori-*associated ITP however; the patients’ platelets did not improve after two courses of triple antibiotic therapy. The patient was treated based on the current guidelines with corticosteroids, IVIG, and splenectomy. Except a temporary spike in her platelet count (Figure 1) after IVIG administration, the patient failed to have a durable response to standard therapy.

After delivery, the patient’s newborn was diagnosed with congenital CMV. While CMV is known to cause secondary ITP, isolated thrombocytopenia, and fetal damage, in most countries pregnant women are not routinely screened for CMV.^11^ Indeed, neither the American Society of Obstetrics and Gynecology (ACOG) nor the Centers for Disease Control and Prevention (CDC) recommend routine serologic screening for CMV in pregnant women.^12^ Furthermore, the current ITP guidelines from the American Society of Hematology do not require CMV testing.^10^ Consequently, medical insurance companies may refuse to cover the costs of CMV testing in patients that have isolated thrombocytopenia and do not exhibit symptoms that suggest CMV. Indeed, in the United States, isolated thrombocytopenia is not considered to be reimbursable indication for CMV testing by Medicare and insurers may refuse payment for asymptomatic patients.

While some experts recommend testing for CMV in thrombocytopenic patients that have lymphocytosis, atypical lymphocytes, toxic granulation or neutrophilia, our patient had none of these signs of CMV infection.^7^ As such, our patient was not tested for CMV during her pregnancy and the diagnosis of CMV-associated ITP was only considered in our patient postpartum, when the fetal urine and placenta tested positive for CMV infection.

The patient’s clinical course and bone marrow findings suggest that the persistent thrombocytopenia was due to secondary ITP caused by CMV infection. We propose that the mother either acquired CMV infection early in pregnancy or had a reactivation of the virus leading to symptomatic CMV causing her to have thrombocytopenia and ITP. Moreover, we hypothesize that the placenta was a reservoir for CMV, thus causing persistent ITP that was refractory to standard therapy, resolving only after delivery. Indeed, mammalian models have demonstrated that the placenta can act as a reservoir for CMV long after it is cleared from the maternal blood.^13^ Soon after the delivery, the CMV reservoir was removed, and maternal platelet count returned to normal levels.

Cytomegalovirus infection in an immunocompetent host is frequently asymptomatic and detected retrospectively.^14^ Increasing evidence from the recent literature suggests that CMV infection may be associated with ITP, especially refractory ITP.^2^ A study by Sheng Yu et al. showed that CMV infection, especially the gB1 genotype, was the cause of ITP in children.^15^ A recent review by DiMaggio et al., suggests that CMV may be the cause of severe and refractory ITP and that CMV PCR should be ordered if there is a strong clinical suspicion of an infection.^6^,^16^ To date, 9 cases of severe, steroid-resistant CMV-associated thrombocytopenia and ITP have been reported (Table 1) in immunocompetent adults.^2^–^6^,^17^–^20^ In some of the cases, thrombocytopenia associated with CMV-induced ITP worsened after administration of steroid therapy.^6^,^15^

Although the mechanism of CMV-induced thrombocytopenia is unclear, several hypotheses have been proposed. Molecular mimicry leading to the production of anti-platelet antibodies causing immune dysregulation and platelet destruction is one such mechanism.^21^ Additionally, CMV directly infects megakaryocytes leading maturation arrest causing decreased platelet production and thrombocytopenia.^21^ Indeed, this has been described as the cause of delayed platelet recovery following allogeneic bone marrow transplant.^22^ Furthermore, the latter better explains the lack of a durable response with corticosteroids, as this therapy is aimed at blocking platelet destruction and not increasing platelet production.^23^ Accordingly, in non-pregnant patients, a trial of a thrombomimetic agent may be useful in severe refractory cases of ITP. Moreover, treatment with ganciclovir and Cytogam to optimize rapid suppression of CMV may provide greater efficacy and faster clinical improvement. Indeed, treatment with anti-CMV therapy has produced a rise in platelet count and improvement in clinical outcome in some patients with CMV-associated ITP.^5^,^6^,^19^,^20^

The current case and literature reports (Table 1) suggest that CMV-associated ITP can cause severe thrombocytopenia refractory to standard therapy and treatment of the underlying CMV infection may improve thrombocytopenia. However, while some clinicians check for CMV in patients with thrombocytopenia, the current American Society of Hematology (2011) guidelines for the diagnosis and management of ITP recommend checking and treating only for Hepatitis C and HIV; they do not recommend testing for CMV as part of the diagnostic workup, representing a gap in the current guidelines.^10^ We believe it is appropriate to test for CMV infection if there is a high clinical suspicion of CMV exposure, steroid-dependent ITP or before splenectomy. Moreover, treatment with ganciclovir and Cytogam should be further investigated in non-pregnant individuals where CMV-associated ITP is suspected. Accordingly, modification of the American Society of Hematology ITP guidelines and those of other organizations may be warranted. Those modifications should include recommendations for CMV testing in pregnancy and prior to splenectomy since treatment of CMV may improve ITP and eliminate the need for invasive surgery. In addition, this would lead to increase physician awareness of CMV-induced ITP and may encourage reimbursement for CMV testing in patients with isolated ITP, who lack other signs and symptoms of CMV infection. Lastly, clinical trials are needed to investigate the risk-benefit of treating CMV-induced ITP with anti-viral agents in pregnant and non-pregnant individuals.

## Figures and Tables

**Table 1:** Cases of Steroid-Resistant Thrombocytopenia and ITP associated with CMV infection in immunocompetent adults.

*Cases of Cytomegalovirus-Associated Steroid-Resistant Thrombocytopenia and ITP in Immunocompetent Adults*					
Source	Age (yr)	Sex	Platelet Count (×10^9^/l)	Diagnostic Test	Treatment and Outcome
Sugioka et al., 2012^4^	30	M	10	Serum PCR (titers 310 copies/10^6^ cells)	Prednisone (85 mg/day) with no response. Responded to IVIG (0.4 g/kg/day)
DiMaggio et al., 2009^6^	50	M	5	Urine cultures and serum PCR	No response to IV methylprednisolone, IV anti-D, IVIG, or IV vincristine.
	72	M	1	Serum PCR, titers 111 copies/10^6^ cells	No response to prednisone or IVIG. Responded to ganciclovir (5 mg/kg, i.v.) twice daily and cytogam twice weekly
Von Spronsen and Breed, 1996^5^	63	F	5	CMV IgM	Dexamethasone (8 mg/d) with no response. Platelet count improved with ganciclovir (5 mg/kg, i.v.) twice daily.
Sahud and Bachelor, 1978^3^	21	M	12	CMV viral titers (1:1024)	Refractory to high-dose steroids. Improved after splenectomy.
Shimm et al., 1980 17	20	M	<10	CMV culture in blood	Refractory to high-dose steroids; Platelet count improved after splenectomy.
Alliot and Barrios, 2005^2^	80	M	8	CMV IgM	Methylprednisolone (120mg/day) and prednisone (60 mg/day) with minimal response; patient improved with IVIG (1g/kg/day).
Shrestha et al., 2014^20^	22	M	6	Not Described	Failed therapy with steroids and IVIG. Splenectomy did not improve platelet count. Improvement was seen when treated with foscarnet and valganciclovir.
Gural et al., 1998^18^	27	M	2	CMV IgM and IgG; spleen tissue PCR	Hydrocortisone (300 mg/day) and IVIG (1g/kg) with no response. Improvement after splenectomy.
Arruda et al., 1997^19^	34	M	12	CMV IgM and IgG	Prednisone (1–2 mg/kg/day) with minimal response. Recovery with ganciclovir 5 mg/kg twice daily × 21 days.
